# Long-term statin therapy is associated with severe coronary artery calcification

**DOI:** 10.1371/journal.pone.0289111

**Published:** 2023-07-27

**Authors:** Kyari Sumayin Ngamdu, Dhairyasheel S. Ghosalkar, Hojune E. Chung, Jared L. Christensen, Cadence Lee, Celia A. Butler, Tiffany Ho, Alice Chu, Jacob R. Heath, Muhammad Baig, Wen-Chih Wu, Gaurav Choudhary, Alan R. Morrison

**Affiliations:** 1 Departments of Research and Medicine, Vascular Research Laboratory, Providence VA Medical Center, Providence, Rhode Island, United States of America; 2 Department of Research, Ocean State Research Institute, Inc., Providence, Rhode Island, United States of America; 3 Department of Medicine, Section of Cardiology, Alpert Medical School of Brown University, Providence, Rhode Island, United States of America; Nanjing Drum Tower Hospital: Nanjing University Medical School Affiliated Nanjing Drum Tower Hospital, CHINA

## Abstract

**Background:**

Atherosclerosis and consequent risk of cardiovascular events or mortality can be accurately assessed by quantifying coronary artery calcium score (CACS) derived from computed tomography. HMG-CoA-reductase inhibitors (statins) are the primary pharmacotherapy used to reduce cardiovascular events, yet there is growing data that support statin use may increase coronary calcification. We set out to determine the likelihood of severe CACS in the context of chronic statin therapy.

**Methods:**

We established a retrospective, case-control study of 1,181 U.S. veterans without coronary artery disease (CAD) from a single site, the Providence VA Medical Center. Duration of statin therapy for primary prevention was divided into 5-year categorical increments. The primary outcome was CACS derived from low-dose lung cancer screening computed tomography (LCSCT), stratified by CACs severity (none = 0; mild = 1–99; moderate = 100–399; and severe ≥400 AU). Statin duration of zero served as the referent control. Ordinal logistic regression analysis determined the association between duration of statin use and CACS categories. Proportional odds assumption was tested using likelihood ratio test. Atherosclerotic cardiovascular disease (ASCVD) risk score, body mass index, and CKD (glomerular filtration rate of <60 ml/min/1.73 m^2^) were included in the adjustment models.

**Results:**

The mean age of the study population was 64.7±7.2 years, and 706 (60%) patients were prescribed a statin at baseline. Duration of statin therapy was associated with greater odds of having increased CACS (>0–5 years, OR: 1.71 [CI: 1.34–2.18], *p*<0.001; >5–10 years, OR: 2.80 [CI: 2.01–3.90], *p*<0.001; >10 years, OR: 5.30 [CI: 3.23–8.70], *p*<0.001), and the relationship between statin duration and CACS remained significant after multivariate adjustment (>0–5 years, OR: 1.49 [CI: 1.16–1.92], *p* = 0.002; >5–10 years, OR: 2.38 [CI: 1.7–3.35], *p*<0.001; >10 years, OR: 4.48 [CI: 2.7–7.43], *p*<0.001).

**Conclusions:**

Long-term use of statins is associated with increased likelihood of severe CACS in patients with significant smoking history. The use of CACS to interpret cardiovascular event risk may require adjustment in the context of chronic statin therapy.

## Introduction

Coronary artery disease (CAD) due to calcific atherosclerosis is a major cause of morbidity and mortality worldwide [1]. Measures of increased atherosclerotic plaque calcification have been predictive of coronary artery disease burden, cardiovascular events, and all-cause mortality [2–3]. Moreover, an increasing rate of progression of the calcification in the coronary vasculature has been associated with worsening prognosis and increased adverse events [5–7]. More recently, there has been evidence supporting the association between increased density of calcification in atherosclerotic plaques and more stable disease [8–13]. Pharmacotherapy with 3-hydroxy-3-methylglutaryl coenzyme A reductase inhibitors (statins) has been successful at reducing the risk of cardiovascular events such that statins have become the current standard of care in patients at moderate to elevated cardiovascular risk [14–22]. Statins were initially developed as cholesterol-lowering agents, but multiple studies support that statin therapy reduced the risk of myocardial infarction out of proportion to the lipid-lowering effect [18–21]. These pleiotropic effects may be mediated by other biological pathways, including improved endothelial function, reduced oxidative stress, reduced platelet adhesion and thrombogenicity, and alterations in inflammation [23,24].

While statins impact atherosclerotic plaque lipid burden, the exact effect of statin therapy on coronary artery calcification has been less clear [12,25–28]. Some initial reports suggested that statin use either reduced calcification or slowed the progression of atherosclerotic calcification [25,27,29], while others indicated statin use resulted in little to no change in the progression of calcification [26,30]. More recently, a growing number of studies have demonstrated the impact of statin use to be associated with increasing measures of coronary artery calcification [28,31–33]. For example, while coronary lipid atheroma indices have been shown to decline in individuals taking high-intensity statin, this effect was accompanied by a paradoxical increase in coronary calcium indices [28,31]. Additionally, while patients with diabetes mellitus demonstrated increased atherosclerotic calcification, statin use by patients with diabetes mellitus was associated with even more progressive coronary atheroma calcification [34–36].

In experimental atherosclerosis models, we recently identified a potential mechanism of statin-induced atherosclerotic plaque calcification through inhibition of Rac1 protein isoprenylation and consequent dysregulation of the small GTPase, Rac1 [32]. Here we set out to assess the association between statin use and progressive calcification in humans, by studying the total duration of time on statin therapy and its relationship to the consequent degree of coronary artery calcification in a U.S. veteran population with extensive smoking history but without established cardiovascular disease. We took advantage of low dose lung cancer screening CT (LCSCT) for the clinical application of acquiring coronary calcification data without need for additional radiation exposure. Understanding the impact of statin therapy on atherosclerotic calcification may have important implications in our interpretation of cardiovascular event risk associated with elevated CACS derived from CT.

## Materials and methods

The Providence Veterans Affairs Medical Center (PVAMC) Institutional Review Board (IRB) approved this study and granted a Health Insurance Portability and Accountability Act (HIPAA) authorization waiver and waiver of consent. This study complies with the Declaration of Helsinki, and all patient data were managed in full compliance of the HIPAA regulations. This study was a retrospective analysis of medical records, and all patient data were fully anonymized for analysis. For the purpose of reproducing the results or replicating the study, the anonymized minimal dataset can be accessed as a (S1 File).

This was a single center, case-control study comprised of 1654 U.S. veterans who underwent U.S. Preventive Services Task Force guideline-recommended (*i*.*e*. 55 to 80 years of age, 30-pack-year tobacco smoking history, active or quit < 15 years previously) lung cancer screening CT between October 1, 2013, and July 31, 2014 [37,38]. A total of 384 patients were excluded for prior history of CAD or cerebrovascular accident (CVA). A total of 89 patients were excluded because there were no data regarding statin usage in the U.S. Department of Veterans Affairs (VA) electronic medical record (EMR).

CT examinations did not use electrocardiography gating and were carried out on a 128-slice CT scanner (Siemens Healthcare, Erlangen, Germany), using 120 kV tube voltage and 40 mA tube current, 128×0.6 mm collimation, 0.84 pitch, 0.5-s rotation, 380-mm field of view, 512×512-pixel matrix, and 1.00–1.25 mm image reconstruction thickness. The minimum area required to identify calcification was 0.55 mm^2^. The Agatston method was employed for quantifying coronary artery calcium score (CACS) via a semi-automated imaging workstation with readers blinded to patient data [39]. The kappa for inter-observer agreement of CACS was 0.92 (0.88–0.96).

The VA EMR was searched for detailed patient demographics and medical history. Information collected on cardiovascular risk included age, body mass index (BMI), diabetes mellitus (DM), hypertension (patients with prior diagnosis and/or use of medications to control blood pressure), hyperlipidemia and/or lipid-lowering medication use, self-reported cigarette smoking status (current or former), family history of premature CAD, and chronic kidney disease (CKD). DM was defined as prior diagnosis of DM or treatment with glucose-lowering medication. BMI was defined as weight in kilograms divided by height in meters squared. CKD was defined as estimated glomerular filtration rate (eGFR) of <60 ml/min/1.73m^2^ using the Modification of Diet in Renal Disease (MDRD) equation [40]. From the acquired data, atherosclerotic cardiovascular disease (ASCVD) risk scores were calculated [41,42]. Imputations were performed using a mean-value imputation approach for BMI and ASCVD risk score values that were missing at baseline [43].

CAD was defined as having ≥1 of the following: history of myocardial infarction (MI); history of coronary revascularization with either coronary artery bypass grafting (CABG) or percutaneous coronary intervention (PCI); or abnormalities on cardiac testing (exercise treadmill testing, echocardiography, myocardial perfusion imaging, cardiac computed tomography, or coronary angiography). Cerebrovascular accident (CVA) was defined as a hospital or neurologist report with the diagnosis of either an ischemic stroke or transient ischemic attack (TIA). Each of the included variables in this analysis, except for age, sex, and BMI, were categorized during data processing and management. Race/ethnicity was categorized as Caucasian or non-Caucasian persons.

Through the EMR, data were collected on any statin use before the baseline LCSCT CACS measurements. The duration of time on statin therapy was calculated for each patient using the pharmacy records, which included the total number of days statin was dispensed by the pharmacy to the patient in the time before the baseline LCSCT measurements. The duration of time on statin therapy was expressed in years and modelled as both a continuous and a categorical (0 years, >0–5 years, >5–10 years, and >10 years) variable.

Our primary outcome was CACS. Because CACS did not follow a normal distribution in our study population, this outcome was stratified using previously established categories defined by several large observational cohort studies and current expert consensus statements on CAC scoring (none, 0 AU [reference control]; mild, 1–99 AU; moderate, 100–399 AU; and moderate to severe, ≥400 AU) [44,45].

Stata/SE software package version 17.0 (SataCorp LLC, College Station, Texas) was used to carry out data management and statistical analyses. Results for continuous variables with normal distribution were presented as mean ± standard deviation (SD). Continuous variables without normal distribution were presented as median (interquartile interval). Results involving categorical data were presented as an absolute number (percentage). The *t* test was used to compare two independent groups that demonstrated normal distribution. If groups did not demonstrate normal distribution, the Mann-Whitney *U* test was used for comparison between two groups. The Chi-square test was used for comparison of categorical variables. To assess the association between duration of statin use and different CACS categories, ordinal logistic regression models were employed to model the ordinal multi-category CACS outcome (whereby a 2-tailed *p*-value < 0.05 was considered statistically significant), and all computed confidence intervals (CI) were set at 95% level. The proportional odds assumption was tested using likelihood ratio test and the assumption was not violated (*p* = 0.8322). In our final model, we adjusted for ASCVD risk score, BMI, and CKD.

## Results

**Fig 1:**
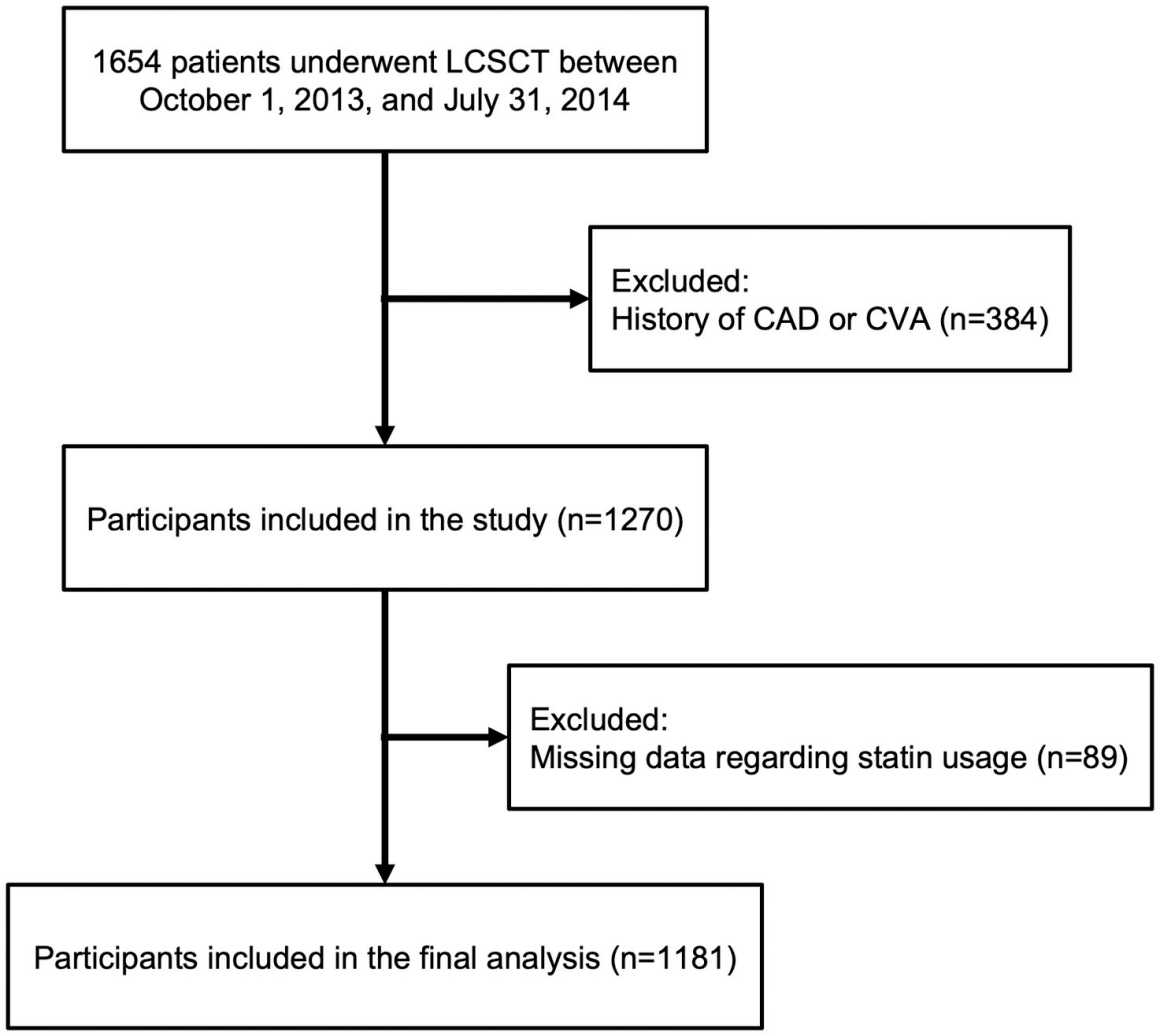
Flow chart of the study population.

We evaluated a total of 1,181 individuals who underwent clinically indicated LCSCT for significant smoking history at PVAMC between October 1, 2013, and July 31, 2014, and met the inclusion/exclusion criteria (Fig 1, Table 1). The mean age of this population was 64.7 (SD: 7.2) years. Indicative of the northeast U.S. veteran population, the vast majority of these patients were Caucasian (94%) and male (95.5%). The mean BMI of the population was 28.9 (SD: 6.1) kg/m^2^. Twenty-five percent of the patients had DM, 56.5% had hypertension, 69% had dyslipidemia, and 12% had an eGFR of <60 ml/min/1.73m^2^. Additionally, 42.5%, 40.9%, and 16.6% of patients were classified as having high, intermediate, and low ASCVD risk score, respectively. Sixty percent of patients had a duration of statin use >0. The overall median CACS was 376.6 AU (IQI: 72.9, 1096.8).

**Table 1:** Baseline characteristics among 1,181 veterans by statin use at baseline.

	Total Population (n = 1181)	No Statin (n = 475)	Statin >0–5 years (n = 425)	Statin >5–10 years (n = 188)	Statin >10 years (n = 93)	p-value
**Age (years)**	64.7 ± 7.2	63.2 ± 7.7	64.7 ± 6.3	67.2 ± 7.3	67.6 ± 6.4	<0.001
**Men**	1128 (95.5)	450 (94.7)	411 (96.7)	180 (95.7)	87 (93.5)	0.40
**Caucasian**	1107 (94)	442 (93.2)	396 (93.6)	178 (94.7)	91 (97.8)	0.37
**BMI, kg/m** ^**2**^	28.9 (6.1)	27.7 (6.3)	29.7 (6.0)	29.7 (5.7)	29.8 (5.5)	<0.001
**DM**	291 (24.7)	50 (10.5)	129 (30.4)	76 (40.4)	36 (38.7)	<0.001
**Hypertension**	667 (56.5)	200 (42.1)	268 (63.1)	129 (68.6)	70 (75.3)	<0.001
**Hyperlipidemia**	814 (68.9)	164 (34.5)	387 (91.1)	175 (93.1)	88 (94.6)	<0.001
**Current smoker**	648 (55)	287 (60.5)	226 (53.3)	92 (48.9)	43 (46.2)	0.007
**Family History of early CAD**	141 (11.9)	53 (11.2)	49 (11.5)	23 (12.2)	16 (17.2)	0.42
**CKD**	139 (11.8)	37 (7.8)	59 (13.9)	25 (13.3)	18 (19.4)	0.002
**Atherosclerotic Cardiovascular Disease (ASCVD) risk score**						<0.001
**High**	324 (42.5)	93 (32.3)	140 (46.5)	57 (48.3)	34 (60.7)	
**Intermediate**	312 (40.9)	118 (41.0)	127 (42.2)	47 (39.8)	20 (35.7)	
**Low**	127 (16.6)	77 (26.7)	34 (11.3)	14 (11.9)	2 (3.6)	

Values are mean ± SD or n (%). BMI = Body mass index; DM = Diabetes Mellitus; CAD = Coronary artery disease; CKD = Chronic kidney disease.

Review of the population stratified by duration of time on statin therapy revealed that patients on a statin for longer periods of time demonstrated a higher prevalence of cardiovascular risk factors (Table 1). Increased duration of statin therapy was associated with slightly older age, increased BMI, and a higher prevalence of DM, hypertension, and hyperlipidemia. Patients with increased duration of statin therapy also had a higher prevalence of CKD and higher ASCVD risk scores. Increased duration of time on statin therapy, divided into 5-year increments, was also associated with increased CACS categories in the unadjusted analyses (Table 2). Further, 2-year increments in duration of statin therapy demonstrated incremental increases in the median CACS of approximately 100–120 Agatston units for every additional 2 years of statin therapy relative to no statin therapy (S1 Fig). Though the majority of participants were Caucasian males, when the sample was stratified by sex and race, there appeared to be no differences in statin use between men and women or white and non-white participants at baseline.

**Table 2:** CACS among 1,181 veterans at baseline.

	Total Population (n = 1181)	No Statin (n = 475)	Statin >0–5 years (n = 425)	Statin >5–10 years (n = 188)	Statin >10 years (n = 93)	p-value
**Total CACS**	377 (73, 1097)	225 (32, 728)	410 (81, 1114)	575 (161, 1383)	972 (400, 1693)	<0.001
**CACS Categories**						<0.001
**0**	121 (10.25)	71 (14.9)	36 (8.5)	12 (6.4)	2 (2.2)	
**1–99**	229 (19.39)	112 (23.6)	87 (20.5)	22 (11.7)	8 (8.6)	
**100–399**	259 (21.93)	120 (25.3)	88 (20.7)	38 (20.2)	13 (14.0)	
**≥400**	572 (48.43)	172 (36.2)	214 (50.4)	116 (61.7)	70 (75.3)	

Values are median (interquartile interval) or n (%). CACS = Coronary artery calcium score.

**Fig 2:**
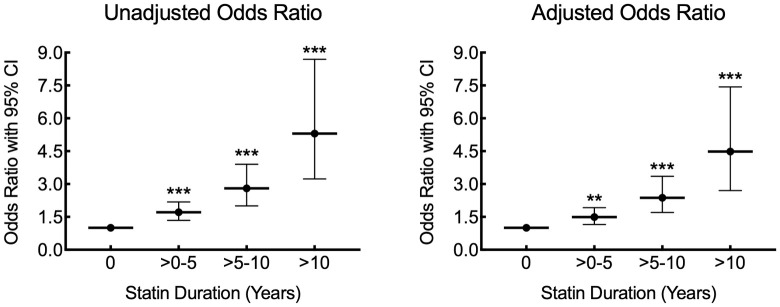
Association between duration of statin therapy and CACS. Graphs of unadjusted and adjusted odds ratios relative to no statin therapy (0). Adjustment included ASCVD risk score, BMI, and CKD (**, P = 0.002; ***, P<0.001 relative to control timepoint 0).

Using duration of statin therapy as a continuous variable in the ordinal logistic regression analysis, increasing duration of statin use (in years) was associated with increasing CACS categories (OR: 1.15 [CI: 1.11–1.18], *p*<0.001), and this association remained significant after the multivariate adjustment (OR = 1.13; 95% CI = 1.09–1.17; p<0.001). There was also a significant association between increasing duration of statin therapy as a categorical variable and increasing CACS category by ordinal logistic regression with no evidence of a threshold effect (>0–5 years, OR: 1.71 [CI: 1.34–2.18], *p*<0.001; >5–10 years, OR: 2.80 [CI: 2.01–3.90], *p*<0.001; >10 years, OR: 5.30 [CI: 3.23–8.70], *p*<0.001) (Fig 2, Table 3). After adjustment for ASCVD risk score, BMI, and CKD, the association between increasing duration of statin therapy and increasing CACS category remained significant (>0–5 years, OR: 1.49 [CI: 1.16–1.92], *p* = 0.002; >5–10 years, OR: 2.38 [CI: 1.7–3.35], *p*<0.001; >10 years, OR: 4.48 [CI: 2.7–7.43], *p*<0.001).

**Table 3:** Relationship between duration of statin therapy as a categorical variable and increasing CACS category.

	Unadjusted			Adjusted		
	OR	(95% CI)	p-value	OR	(95% CI)	p-value
**0 years**	Ref.	—	—	Ref.	—	—
**>0 to 5 years**	1.71	1.34–2.18	<0.001	1.49	1.16–1.92	0.002
**>5 to 10 years**	2.80	2.01–3.90	<0.001	2.38	1.7–3.35	<0.001
**>10 years**	5.30	3.23–8.70	<0.001	4.48	2.7–7.43	<0.001
**ASCVD**	1.06	1.05–1.07	<0.001	1.05	1.04–1.06	<0.001
**BMI**	0.99	0.97–1.00	0.115	0.97	0.96–0.99	0.003
**CKD**	1.43	1.02–2.02	0.038	0.99	0.69–1.42	0.96

ASCVD = Atherosclerotic cardiovascular disease risk score; BMI = body mass index; CKD = chronic kidney disease.

## Discussion

Coronary artery calcification is a marker of the overall burden of atherosclerosis and is associated with worsening cardiovascular events and all-cause mortality [2–4]. However, recent studies indicate that the ultrastructural composition of calcium within plaque may confer features of reduced risk and consequent plaque stability in some instances [8–13]. Statins have been tremendously successful at reducing the risk of cardiovascular events in patients, and this risk reduction has been demonstrated to be out of proportion to the lipid-lowering effect, suggesting alternative mechanisms may help to stabilize coronary plaque [14–22]. While there continues to be some disagreement over whether statins can influence the calcium composition of atherosclerotic plaque, growing clinical data support that statins may increase atherosclerotic calcification [25–33]. Here we find a significant association between duration of statin therapy and total CACS. Specifically, long-term duration of statin therapy was significantly associated with a greater risk of having severe CACS. The increasing odds of having severe CACS being associated with higher duration of time on statin therapy remained significant after adjustment for the baseline cardiovascular risk factors known to significantly impact the CACS (ASCVD risk score, BMI, and CKD).

As mentioned, the effect of statin therapy on CAC has remained controversial to date with some studies showing a decrease or no change in calcification [25–27,29,30]. However, there were several concerns with these studies, including open label design, lack of simultaneous comparison between treatment and control groups, small sample size, and short follow-up duration (<1–2 years) [25–27,29,30]. Our findings appear consistent with a number of preclinical and clinical studies supporting the pro-calcific effects of statins [28,31,32]. Recently, a substudy of the prospective PARADIGM registry revealed baseline statin therapy to be associated with higher total CACS at baseline compared to those that were not prescribed statins and that calcified plaques were more likely to increase in the statin treated group [28]. In the cohort of patients already taking statins, CACS was also correlated with calcified plaque volume and density. However, the statin use was treated as a binary in the analysis and duration of time on statin therapy was not taken into account which could have been an important confounder in the analyses. A recent analysis of two clinical trials indicated that 4–6 years of high-intensity statin therapy was associated with progressive CACS [33]. Our data further strengthens the conclusion of a relationship between statin and CACS in about 1200 patients with extensive smoking history and higher cardiovascular risk, incorporating a longer total duration of time (5–10 and >10 years) on statin therapy prior to the CT. Moreover, we studied a higher cardiovascular risk population, taking advantage of low-dose lung cancer screening CT and the application of non-gated image analysis for calcium scoring.

Statin therapy is well-established as the current standard of care for lipid management and the prevention of cardiovascular events [15–17,22,46]. In fact, statins are so effective at reducing events, current guidelines for cholesterol management have taken an aggressive stance on prescribing statins [22]. However, these same guidelines recommend consideration of CACS to refine risk assessment when statin therapy is problematic or there is a question regarding continuation *vs*. intensification of statin therapy. While CACS independently predicts ASCVD events in a graded fashion, current risk analyses utilizing CACS do not take into account statin use or duration of time on statin therapy. Our study raises questions as to the accuracy of the CACS value for risk assessment in the context of long-term statin use, and our data suggest that modeling of incremental risk associated with CACS may require some degree of adjustment for those patients who are on long-term statin therapy. Though a number of studies suggested that CACS retains predictive value for events [47–49], it is important to note that these studies were carried out in younger populations of patients with much lower CACS, requiring that CACS analysis be treated as binary. Moreover, lipid lowering was either unspecified or also treated as a binary, such that the exact impact of duration of statin therapy was not assessed. More recently, data from the CAC Consortium observational registry suggested that the prognostic significance of CACS may in fact be slightly reduced in statin users, particularly those with higher CACS values, raising important questions about the impact of statin therapy on the components of the Agatston scoring system [50]. Prospective analyses are needed to better quantify this impact of duration statin therapy on the relationship between CACS and outcomes.

We have previously demonstrated a potential mechanistic pathway of statin-induced, Rac1-dependent atherosclerotic vascular calcification [32]. Briefly, we found that statin treatment prevents isoprenylation of Rac1 protein disrupting the complex between Rac1 and its inhibitor, RhoGDI, and leaves Rac1 free to become activated by exchange for guanosine-5’-triphosphate (GTP). In its active form, Rac1 acts by promoting expression of proinflammatory cytokines including IL-1β leading to vascular calcification [32,51–53]. The CANTOS (Canakinumab Anti-Inflammatory Thrombosis Outcome Study) trial demonstrated that inhibition of the potent inflammatory cytokine IL-1β, led to a reduction in secondary cardiovascular events [54]. The overall benefit to all-cause and cardiovascular mortality appeared to be primarily in patients who demonstrated reductions in the systemic inflammation marker high-sensitivity C-reactive protein [55]. The vast majority (93.4%) of patients enrolled in the CANTOS trial were on lipid-lowering therapy, consisting of primarily statins, and thus the trial design is one in which systemic IL-1β inhibition was added to statin therapy. Of note, there have been no data to date regarding CACS or evolution of calcification imaging from CANTOS. It is interesting to speculate whether calcification imaging would be influenced by IL-1β inhibition given the findings in preclinical models.

Limitations to our study include those inherent to the study design as single site, retrospective analysis of primarily Caucasian, male U.S. veterans. Therefore, results are subject to a degree of selection bias and are less generalizable to women and other racial/ethnic groups. Further, our cohort was restricted to veterans who underwent lung cancer screening as recommended by U.S. Preventive Services Task Force guidelines for extensive smoking histories. Key risk factors such as dyslipidemia, hypertension, and diabetes mellitus were captured as binaries in our analyses, and we could not adjust for the degree of control of these conditions in individual patients. We also could not adjust for the original reasons for prescribing statins, and we did not have access to whether low-density lipoprotein cholesterol met target goals with therapy. Moreover, our study makes use of extracted data from the VA Pharmacy’s electronic medical record, and there is a possibility that statin usage outside the Department of Veterans Affairs was not fully captured. Dispensed medication may not fully account for compliance with regards to taking the medication. Finally, the relationship between statin duration, CACS, and cardiovascular events in this cohort was not studied here. Despite these limitations, our findings are consistent with other studies supporting the pro-calcific effect of statin therapy, indicating further study in this area is needed [28,31–33]. These limitations should be addressed in future studies focusing on diverse patient populations, and the future research designs should be expanded to include other factors that can refine the link between statin use, high CACS, and predictive value for outcomes.

## Conclusion

In conclusion, long-term duration of time on statin therapy is associated with likelihood of having severe CACS in patients who have sufficient smoking history to qualify for lung cancer screening. These findings highlight an important complexity to the relationship between statin therapy and CACS, indicating that risk from CACS should be interpreted not just in the context of traditional cardiovascular risk factors and serial CACS progression, but also in the context of plaque-altering treatment. The use of CACS to interpret cardiovascular event risk may require adjustment in the context of chronic statin therapy.

## Supporting information

S1 Fig Increasing 2-year increments in duration of statin therapy demonstrate increasing median CACS. Data were plotted as median CACS with interquartile range. (N) indicates the number of patients in each additional 2-year increment in duration of statin therapy. *, P = 0.01; **, P<0.005; ****; P<0.0001, compared to duration of statin “0” using Kuskal-Wallis followed by Dunn’s multiple comparison testing.
(TIF)

S1 File Fully anonymized minimal data set. (XLS)
