# Supplementary figures and images for: Long-term statin therapy is associated with severe coronary artery calcification
Source: PLoS One. 2023 Jul 27;18(7):e0289111. doi: 10.1371/journal.pone.0289111 (PMC10374064; doi:10.1371/journal.pone.0289111)

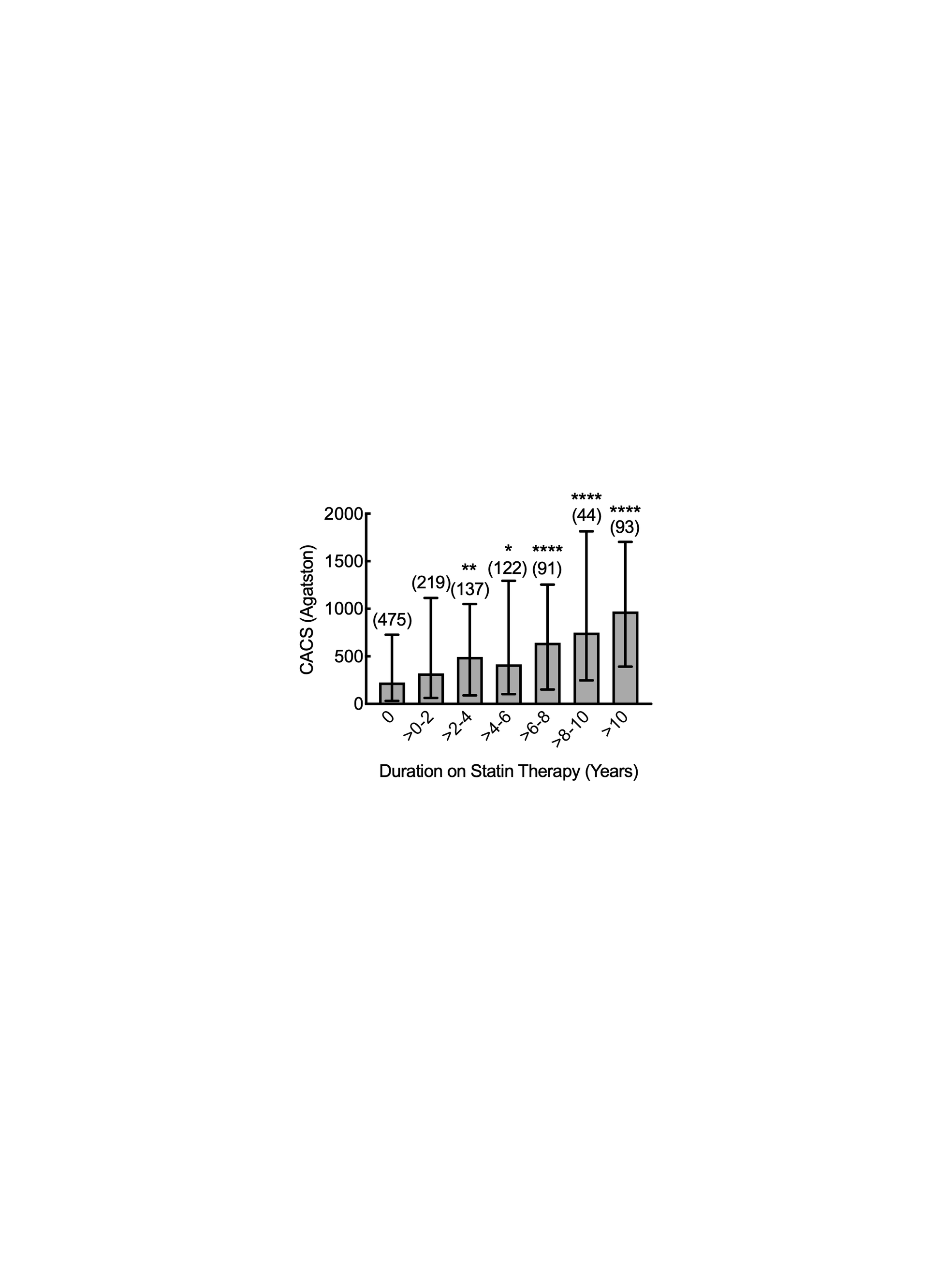

Supplement: S1 Fig — Data were plotted as median CACS with interquartile range. (N) indicates the number of patients in each additional 2-year increment in duration of statin therapy. *, P = 0.01; **, P<0.005; ****; P<0.0001, compared to duration of statin “0” using Kuskal-Wallis followed by Dunn’s multiple comparison testing. (TIF) [file pone.0289111.s001.tif]
